# Mirror invariance dies hard during letter processing by dyslexic college students

**DOI:** 10.1038/s41598-025-21092-5

**Published:** 2025-10-27

**Authors:** Tânia Fernandes, Mariona Pascual, Susana Araújo

**Affiliations:** CICPSI, Faculty of Psychology, Universidade de Lisboa, Lisbon, Portugal

**Keywords:** Visual word recognition, Developmental dyslexia, Mirror invariance, Mirror image, Orthographic processing, Masked priming, Psychology, Human behaviour

## Abstract

Reversal errors (e.g., confusing b with d, or R with Я) are common in beginning readers and often persist in individuals with developmental dyslexia due to *mirror invariance*—an evolutionary-old perceptual tendency of processing mirror images as equivalent. This study investigated whether dyslexic adults still struggle with mirror-image discrimination when processing *reversible* letters (i.e., differing only by orientation; e.g., d, b, p) and *nonreversible* letters (i.e., differing also in shape; e.g., f, t, r). In a masked priming lexical decision task, one letter of the prime was manipulated by *letter-type* (reversible, nonreversible) and *prime-condition*: identity (e.g., judo, zero), control (judo, zero), mirrored-letter (jubo, zero), or rotated-letter (jupo, zero). Both dyslexic and neurotypical readers showed *identity priming* effects: faster recognition of target-words preceded by identity than control primes. Neurotypical readers also showed *mirror* and *rotation costs*, regardless of letter-type: slower word recognition after mirrored- or rotated-letter primes than an identity prime. In contrast, and for nonreversible letters only, dyslexics were as fast in recognizing target-words preceded by identity as by mirrored-letter primes (qualified by Bayesian statistics). These findings suggest that, despite extensive reading experience, orthographic processing by dyslexic college students remains residually sensitive to mirror invariance.

**Supplementary Information:**

The online version contains supplementary material available at 10.1038/s41598-025-21092-5.

## Introduction

Reading is a major cultural achievement, supported by a specialized brain network that is remarkably consistent across culture, script, and age of literacy acquisition (for reviews, see^1,2^). The left *ventral occipitotemporal* cortex (vOT) is a core region of this network, underpinning the *orthographic* system—the “mid-level vision of reading^3^”—responsible for transforming visual input, from pixels into abstract letter identities and into written word forms^4,5^. Notably, when processing print, hypoactivation of the vOT has been consistently found in readers with *developmental dyslexia*^6–9^*,* i.e., a neurocognitive disorder that impedes reading development despite no general learning problems or sensory deficits and appropriate educational settings and motivation to learn^10^.

The reproducibility of the reading brain network–particularly of the neural substrates of orthographic processing–relies on *recycling* of part of the *ventral visual* stream originally dedicated to object recognition^1,2^. As a result, the orthographic system inherits the perceptual biases of object recognition, including those that may hinder automatic letter and visual word recognition, such as mirror-image generalization or *mirror invariance*^1,11,12^. For example, after training on a novel item in a given orientation (e.g., ‘d’ or ‘r’), observers tend to perceive its *mirror image* (e.g., ‘b’ or ‘ɹ’) as perceptually equivalent, while not generalizing across other orientation contrasts like *plane rotations* (i.e., rotations in the picture plane; e.g., ‘d’ vs. ‘p’, or ‘r’ vs. ‘ɹ’)^12–14^. However, for the sake of efficient letter and word recognition, mirror invariance cannot be tolerated in scripts with *reversible* letters (i.e., with the same shape as other letters but which differ by orientation only, e.g., d, b, p) as well as *nonreversible* letters (i.e., which differ from other letters both in shape and orientation, e.g., r, t, f). Otherwise, words like *dig*, *big*, *did*, *bid* would be hard to discriminate. In this study, we investigated whether mirror invariance “dies hard” during letter processing in developmental dyslexia, examining whether this evolutionary-old perceptual bias inherited from object recognition, still influences letter identity coding during visual word recognition in dyslexic college students.

Mirror-image discrimination is especially triggered when learning scripts that contain mirrored reversible symbols, like the Latin alphabet^15–17^. Recent studies adopting the *masked priming* paradigm—a gold standard used to investigate the orthographic code (for a review, see^18^)—have shown that mirror invariance no longer operates during orthographic processing in fluent adult readers^19–22^ (for converging neuroimaging evidence, see^23,24^). This does not imply that mirror invariance is inhibited early on in orthographic processing; rather, mirror-image discrimination occurs automatically at a prelexical stage of processing^19–21^.

In masked priming studies, a brief prime (30–60 ms) is masked because it is presented in-between a hash string (e.g., #####) and a target (e.g., ZERO), rendering the prime subliminal while still influencing target recognition. This paradigm is thus resistant to strategic factors and taps into letter coding and early orthographic processes^3,25,26^. Target identification is maximally facilitated in the *identity* prime condition, where the prime corresponds to the target, regardless of cross-case similarity (e.g., ‘judo’ as a prime for ‘JUDO’)^27^. Prelexical orthographic processing is typically investigated by also including *form* primes, i.e., letter strings that share most letters with a target (e.g., ‘jupo’ for ‘JUDO’). Notably, the lexical status of a prime (for target words, a word in the identity condition and a nonword in the form condition) does not affect the magnitude of the effects found in lexical decision (for direct evidence, see^28^). Critically, the degree of lexical activation driven by the prime depends on the precision of letter identity coding. Thus, comparing identity and form primes allows investigating whether letter identity coding remains sensitive to the perceptual biases inherited from object recognition. If mirror invariance still operates, then a form prime like ‘jubo’ would activate the word ‘JUDO’ as effectively as an identity prime. Likewise, primes containing nonletters that are visually similar to letters (e.g., ‘1D34’ or ‘!D€Δ’ instead of ‘IDEA’) yield facilitation relative to visually dissimilar primes (e.g., ‘7D26’ or ‘?D% □’)^29,30^. This is because such nonwords, including those with nonletters, engage early, prelexical processing mechanisms that are attuned to operate on visually ambiguous input^3,5,18,26^.

Fernandes et al.^19^, like Perea et al.^22^, examined mirror-image processing of reversible and nonreversible letters with the masked priming paradigm (for separate evidence on nonreversible and reversible letters, see^20,21^). Fernandes et al. capitalized on the fact that the Latin alphabet comprises both mirrored and rotated reversible letters (e.g., d and b, d and p). This is an important property of this script because both types of orientation contrast have the same angular difference, similar pixel-level overlap, and share features and geometric shape – making them *perceptually similar*–yet mirror invariance applies only to mirror images^12–14^. When one letter in the prime was replaced by an *orientation transformation* (either a mirror-image or a plane-rotation), visual word recognition (e.g., ‘IDEA’, ‘ZERO’) was significantly slower after both *mirrored-* and *rotated-letter* primes (e.g., ‘ibea’ and ‘ipea’; ‘zero’ and ‘zeɹo’) compared to identity primes (e.g., ‘idea’, ‘zero’). These orientation costs relative to identity primes cannot be attributed to heightened inhibition of mirrored letters due to mirror invariance^22^, but rather to perceptual similarity allied with the interactive activation dynamics of the orthographic system^4,31^, which hold for both mirror images and plane rotations.

The identity prime condition provides the appropriate reference baseline because it differs from orientation-transformed primes only in the critical dimension of interest (for a similar rationale, see^32,33^). It thus allows estimating the processing cost associated with a change in letter orientation. A significant *mirror cost–*i.e.*,* slower responses after mirrored-letter primes than identity primes–indicates automatic mirror-image discrimination at a prelexical stage, whereas equivalent performance between these prime conditions is consistent with mirror invariance.

Critically, mirrored and rotated transformations are orientation contrasts that can apply to any visual stimulus, including nonreversible letters for which such transformations result in nonletters. For reversible letters, these orientation transformations correspond to other (real) letters, triggering activation of multiple letter representations that then inhibit each other^4,31^. In contrast, orientation transformations of nonreversible letters (e.g., ‘f’ vs. ‘ɟ’) do not result in other existing letters, and thus, mirror invariance could, in principle, still be observed^20,22^. However, fluent adult readers showed a significant mirror cost even for nonreversible letters, indicating that mirror invariance is no longer at play during orthographic processing^19,20^. Both mirrored-letter (e.g., soɟa) and rotated-letter (e.g., soɟa) primes led to slower word recognition than identity primes (e.g., sofa) because letter identity is defined by both shape and orientation^17^. The orientation transformations of nonreversible letters activate mostly a single letter node, albeit less than that elicited by the canonical form^4^. Automatic mirror-image discrimination by fluent adult readers when processing reversible or nonreversible letters is robust. It has been found across independent studies in different alphabetic languages^19–22^, with various tasks (e.g., same–different matching; lexical decision^19,21^), and different masked priming variants (e.g., conventional, sandwich priming^20,22^).

Likely because mirror invariance is an evolutionary legacy^1,11–14^, automatic mirror-image discrimination takes time to develop and depends on the formation of abstract letter representations. Fernandes et al.^34^ showed that in typically-developing 2nd-4th-grade beginning readers, mirror invariance still operates during orthographic processing. These children showed significantly faster visual word recognition (e.g., ‘ALBUM’; ‘ARENA’) after an identity prime (e.g., ‘album’; ‘arena’) than a control prime (e.g., ‘al░um’; ‘a░ena’). This identity priming effect demonstrates that lexical activation and access were already in place in these young readers. More important, there was no hint of a mirror cost for either reversible or nonreversible letters as mirrored-letter primes (e.g., ‘aldum’; ‘aɹena’) facilitated word recognition just as much as identity primes, whereas rotated-letter primes (e.g., ‘alqum’; ‘a﻿ɹena’) did not. At the individual level, the emergence of mirror-image discrimination was predicted by the quality of letter representations, not by age or phonological skills. By 5^th^-grade, mirror-image discrimination emerged for reversible letters, and it only extended to nonreversible letters by the end of 6th-grade, marking the full transition to an adult-like orthographic system^34^.

It remains to be tested whether dyslexic readers fail to automatize mirror-image discrimination during orthographic processing, as suggested by previous studies^11,35–38^ (for a review, see^39^). Since automatic mirror-image discrimination develops slowly in typical readers and depends on the quality of abstract letter representations^34,38,40^, its trajectory in dyslexia is difficult to chart. Two factors have further complicated this picture: (1) methodological confounds in previous research, and (2) the persistence of orthographic deficits in dyslexic adults.

On the one hand, the tasks and age groups used to investigate mirror-image processing in dyslexia raise concerns. Most studies have adopted same-different matching or letter-naming tasks^35,37–41^, which do not predominantly engage orthographic processing nor represent the best choice for investigating letter coding (for discussion, see^21,34^). Additionally, most studies have examined mirror-image processing in dyslexic under the age of 13 (typically 9–12 years old)^35–39,41^, who often lag 18–24 months behind their peers in reading level^10^. It is thus unsurprising that they remain sensitive to mirror invariance, especially for nonreversible letters, given that typical readers below Grade 6 show this same pattern^34^. In other words, deviant mirror-image processing in dyslexia may reflect impoverished^42^ or suboptimal^43^ print exposure and limited orthographic experience. This confound can only be ruled out by testing dyslexic adults with extensive reading practice, like dyslexic college students, who are often characterized by slow but accurate reading^44,45^.

On the other hand, orthographic deficits in dyslexic adults cannot be attributed solely to insufficient or limited reading experience^45,46^. Their letter representations remain atypical at both behavioral^37,38,47,48^ and brain^6–8^ levels, suggesting a core disruption in orthographic processing. As a result, even in adulthood, orthographic processing in dyslexia could remain more vulnerable to the perceptual biases of object recognition. For example, dyslexic college students, but not controls, are susceptible to the visual contour of words during reading^49^. Similarly, 11-years-old dyslexics–but not typical readers–also show greater difficulty in lexical decision on nonwords that visually resemble real words (e.g., more errors on ‘viotin’ than on ‘viocin’, due to similarity with ‘violin’)^50^.

Very few studies examined mirror-image processing in dyslexic adults (not limited to college students), but the findings suggest that mirror invariance could still operate during letter processing. In a serial letter naming task^51^, dyslexic college students’ naming and eye movements were disproportionally more affected by the presence of mirrored reversible letter pairs than controls. Similarly, when presented with a colored-letters matrix^44^, on which participants were asked to rapidly switch from naming a letter to naming a color, dyslexic college students showed greater interference (in gaze duration and eye-voice span) when a reversible letter was preceded by its mirrored counterpart than by a visually dissimilar letter, whereas controls did not. Given that there were no group differences in a baseline condition, the difficulty seems specific to mirror images. In a letter-sound matching task^52^, both groups performed similarly on congruent and phonologically similar but incongruent letter-sound pairs. However, only dyslexics made more errors on mirrored letter pairs. Finally, in a same-different task on word pairs^47^, dyslexic adults made more errors when the pair differed by a mirrored letter (e.g., cod—cob) than by a visually similar letter (e.g., fire—tire), whereas controls did not. Only Peter et al.^47^ directly compared mirrored letters and other visually similar letters, which is crucial for isolating the role of mirror invariance. However, all these studies used naming or same-different tasks, raising the methodological confounds aforementioned.

The present study addressed these shortcomings by adopting a masked priming lexical decision task to assess whether mirror invariance still operates in high-functioning dyslexic adults when processing reversible and/or nonreversible letters. It builds upon previous work using the same materials, paradigm, and task–validated in both children and fluent adult readers^19,34^. Dyslexic and control college students were presented with uppercase target words with reversible or nonreversible letters (e.g., ‘JUDO’, ‘ZERO’) preceded by lowercase primes in one of four prime conditions: identity (e.g., ‘judo’, ‘zero’), control (with one letter replaced by a dot-pattern, e.g., ‘ju░o’, ‘ze░o’), mirrored-letter (e.g., ‘jubo’, ‘zeɹo’), or rotated-letter (e.g., ‘jupo’, ‘zeɹo’).

We first checked for the identity priming effect (identity vs. control primes) to ensure successful lexical access in both groups. Next, we examined the orientation costs for mirrored and rotated primes relative to identity primes. Given the previous findings with typical readers^19–21,34^, we predicted that, if mirror-image processing remained deviant in dyslexic adults, then either a Group x Prime or a Group x Letter x Prime interaction would be found. The former interaction would indicate persistent mirror invariance across letter types in dyslexics, whereas the latter would suggest that mirror invariance is specific to nonreversible letters. We also computed an independent, standardized measure of the mirror cost unaffected by overall response speed, that is, *Cohen’s d*^19,34,53^. Additionally, Bayesian statistics^54,55^ allowed quantifying the evidence for equivalence in cases where Null-Hypothesis Significance Testing (NHST) yielded null effects, since mirror invariance in dyslexics would correspond to equivalent performance in identity and mirrored prime conditions.

## Methods

### Participants

An a-priori power analysis with mixedpower^56^ on R^57^ indicated that a sample size of 15 per group would allow detecting a significant Letter x Prime interacion in each group with an effect size of η_p_^2^ = 0.20 and 48 items per condition, with a power of 0.80 and a = 0.05 (cf. Fernandes et al.^19^, Experiment 1).

Two groups of Portuguese native-speaking college students (19–29 years old; *M*_*age*_ = 23.47, *SD* = 2.47), with normal or corrected-to-normal vision and no known neurological or psychiatric disorders, participated voluntarily after they gave written informed consent. The dyslexic group included 18 participants (13 women, 5 men) with a formal clinical diagnosis of developmental dyslexia, no comorbid disabilities, and self-reported reading difficulties. All showed current reading performance indicative of persistent reading difficulties in two reading tests: (1) the 1-min TIL test^58^, a text comprehension screening test and the only standardized instrument with normative data available for Portuguese college students, and (2) the reading fluency test of the Differential Diagnosis Dyslexia Battery, 3DM (Portuguese version^58^). The control group consisted of 20 neurotypical adults (13 women, 7 men) with no history of developmental disorders or reading complaints. The groups were matched for age, *t*(36) = −0.32, *p* = 0.75, *Cohen’s d* = −0.10, BF_01_ = 3.04, schooling (i.e., last grade successfully completed), *t*(36) = −0.25, *p* = 0.80, *Cohen’s d* = −0.08, BF_01_ = 3.09, and sex, *X*^*2*^_(1)_ = 0.23, *p* = 0.63.

As shown in Table 1, the groups were also matched in nonverbal intelligence (Raven’s Standardized Progressive Matrices, RSPM^59^) and visuospatial working memory (Corsi-block test^60^), but differed significantly in reading skills. None of the controls scored below the cutoff on the 1-min TIL, a screening test for reading problems^61^. Although the 1-min TIL is formally a text comprehension test, it is highly saturated in phonological decoding, sharing 78% of variance with the 3DM reading fluency test via this common latent factor ^61^. Therefore, given the 1-min TIL scores of control readers, the absence of reading complaints, and significantly more reading fluency than the dyslexic group (Table 1), it is unlikely that any control participant had undetected difficulties of reading fluency.

**Table 1:** Characterization and comparison of the control and dyslexic groups.

	Controls (n = 20)	Dyslexics (n = 18)	Comparison and *Cohen’s d*
RSPM^a^	11.80 (2.15) [7–16]	11.11 (2.56) [7–16]	*t*(36) = 0.89, *p* = .38 and *0.29*
Corsi block^b^	15.70 (3.42) [13–25]	16.94 (3.55) [8–23]	*t*(36) = −1.10, *p* = .28 and *−0.36*
1-min TIL^c^	16.60 (2.74) [13–24]	10.22 (2.88) [6–15]	***t*(36) = 6.99, *p* < .001 and *2.27***
3DM reading fluency^d^			
High-frequency words	62.60 (6.74) [51–75]	47.22 (9.97) [26–62]	***t*(36) = 5.62, *p* < .001 and *1.83***
Low-frequency words	57.50 (9.11) [44–74]	38.50 (9.60) [15–51]	***t*(36) = 6.26, *p* < .001 and *2.03***
Pseudowords	44.80 (7.94) [34–61]	29.22 (8.99) [7–42]	***t*(36) = 5.67, *p* < .001 and *1.84***

Mean scores; *SD* are in parenthesis. Minimum and maximum scores are in brackets. Cohen’s *d* is in italics. Significant results are in bold (two-tailed test; a = 0.05). ^a^ RSPM, Raven’s Standardized Progressive Matrices: Nonverbal IQ and visuospatial skills, standardized score (mean: 10). ^b^ Visuospatial working memory: total number of sequences correctly produced. ^c^ Reading screening test, text comprehension: total number of correct responses (of a maximum of 36). ^d^ Reading fluency: total number of items correctly read aloud per list in 30 s (of a maximum of 75).

This study was approved by the Deontological Committee of Faculty of Psychology, Universidade de Lisboa, Portugal. It was conducted in accordance with internationally recognized standards, including the Declaration of Helsinki, and the Portuguese official regulation for ethics in research in Psychology. Participants were compensated for their time and travel expenses.

### Material and procedure

**Fig. 1:**
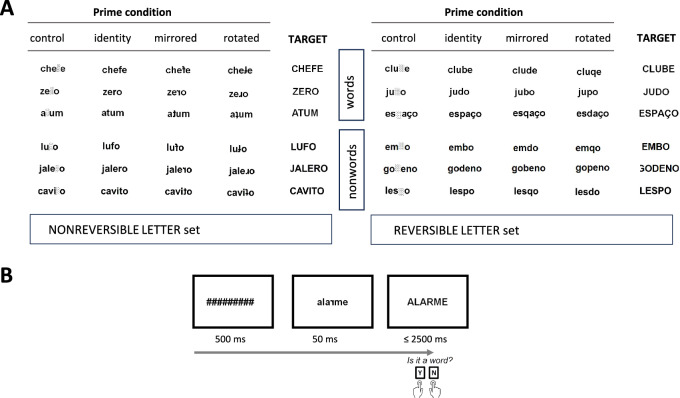
Illustration of the material and trial. (**A**) Examples of words and nonwords used as primes and targets by letter (nonreversible-letter set: f, r, t; reversible-letter set: b, d, p). (**B**) Sequence and duration of events in each trial (the example presents a target word preceded by a rotated-letter prime; English translation: alarm).

Material and apparatus of the lexical decision task were identical to those of Fernandes et al. ([19] Experiment 1) and are illustrated in Fig. 1. Two sets of items (reversible-letter and nonreversible-letter sets) were used, each containing 192 Portuguese words and 192 nonwords (i.e., phonotactically and orthotactically legal nonwords used as fillers in the lexical decision task); in total, 384 words and 384 nonwords, previously validate in a study with children^34^.

As illustrated in Fig. 1A, the reversible letter set comprised words and nonwords with a critical reversible letter (e.g., critical letter underlined:, CLUBE, JUDO, ESPAÇO; English translation: club, judo, space, respectively; in the nonwords, EMBO, GODENO, LESPO) and the nonreversible letter set comprised words and nonwords with a critical nonreversible letter (e.g., in the words, CHEFE, ZERO; ATUM; English translation: chief, zero, tuna, respectively; and in the nonwords, LUFO, JALERO, CAVITO).

As detailed in previous studies^19,34^, the two sets were carefully matched in visual and linguistic dimensions. Both used asymmetrical consonants, that is, *b*, *d*, *p* (reversible), and *f*, *r*, *t* (nonreversible), to ensure that mirrored and rotated transformations differed from one another and from the upright canonical form. Additionally, a pretest conducted by Fernandes et al.^19^ ensured that mirrored and rotated transformations were equally confusable with the canonical form for both letter types, and that their perceived similarity was comparable across reversible and nonreversible letters. The reversible letters were specifically chosen because both their mirrored and rotated transformations correspond to real letters, given that both were used as primes in this study. The two sets were matched for cross-case similarity^62^.

Furthermore, the two word and nonword sets (reversible-letter and nonreversible-letter sets) were also carefully matched on length, position of the manipulated letter (on average, the third), and distribution of manipulated letters within each set (48 items for b/f; 48 items for p/t; 96 items for d/r). This ensured than no systematic bias related to letter position or string structure could confound the results. Each set comprised 4–9 letters long (2–4 syllables) items, and hence, length was not fixed nor manipulated, as the two sets were matched for it, but items were of varied lengths to ensure generalizability of the findings to polysyllabic items in general, rather than being restricted to a fixed word length. This approach is common in masked priming studies, including in those investigating mirror-image letter processing (e.g.,^20–22^).

The two sets of words were familiar and likely well-known, as these words are well-attested in primary school textbooks^63^, and 73% already appear in written form in first-grade textbooks^34^. As shown by Fernandes et al.^19,34^, these word sets were matched in word frequency (both on adult and child corpora: CORLEX—available at https://clul.ulisboa.pt/projeto/lexico-multifuncional-computorizado-do-portugues-contemporaneo and ESCOLEX^63^), neighborhood size, number of neighbors differing from the target-word in the critical letter, and orthographic and phonetic uniqueness points. The corresponding nonword sets were also matched in length and average number of lexical neighbors. The full item list is available as Supplementary Material in Fernandes et al.^19^, and the psycholinguistic properties of each item are publicly available at https://osf.io/bdpq8/.

As shown in Fig. 1A, for each letter string, an uppercase version was used as target. The identity prime was the lowercase version of the target, from which the other prime conditions differed only in the critical letter. In the control prime, it was replaced by a dot-pattern; in the mirrored prime, by the letter’s mirror-image; and in the rotated prime, by the letter’s 180º plane-rotation.

Participants were tested at the experimental laboratory of the faculty. They first completed the ancillary tasks reported in Table 1, followed by a lexical decision task; they sat at ~ 60 cm from the monitor (resolution: 1024 × 768 pixels; refresh rate: 60 Hz). Timing, sequence of events, and data collection (accuracy and reaction time, *RT*, from target onset) were controlled by E-Prime SP1. The sequence and duration of events in each trial is presented in Fig. 1B. Participants were asked to decide as quick and accurate as possible whether the uppercase target was a Portuguese word or not, using two designated keys (*yes*, word response; *no*, nonword response).

For familiarization, participants first completed 16 practice trials (8 words and 8 nonwords) and received feedback on accuracy of their responses. Next, they performed two blocks of 384 experimental trials each, with order randomized, and a self-paced break between blocks (no feedback was provided on experimental trials). Four lists were created to counterbalance the four prime conditions. Each participant was presented with one list, encountering each item once (48 items per condition). Across lists and between participants, each item appeared in all four prime conditions.

### Statistical analysis

The raw data (RT and accuracy) in each trial for each participant and the analyses code are publicly available at https://osf.io/z38w9.

For the sake of completeness, performance on nonword trials (errors and RTs for correct responses) is reported in Supplementary Material, Table S1, but not analyzed, as nonword masked priming effects are usually unreliable in lexical decision^18^. An analysis of errors in word trials is reported in Supplementary Material, Table S2, to rule out speed/accuracy trade-offs. Our primary dependent variable was the RTs for correct word responses, trimmed by excluding those differing by at least 2.5 *SD* from the grand mean of each participant. Next, two types of independent analyses were conducted on these raw RTs:

*First*, we fitted a *linear mixed-effect model* (LMM) to log RTs of correct word responses (to ensure no violation of LMM assumptions) using R^57^, with the lme4^64^, lmerTest^65^, and the bobyqa optimizer of afex^66^. Group, Letter, and Prime were fixed factors (all factors centered, adopting sum coding), and by-subjects and by-items random intercepts were included (formula: lmer(log(RT) ~ Group * primeC * letterC + (1|Subject) + (1|Item), data = RT_word, control = lmerControl(optimizer = ‘bobyqa’)). The random-effects structure was chosen based on previous studies^19,34^ and for the sake of statistical power^67,68^. *P*-values were derived using Satterthwaite approximations (REML estimation^69^). LMMs are recommended to properly account for both subjects’ and items’ sources of variance. They overcome the limitations of traditional ANOVAs by avoiding the need for data aggregation (which can obscure important sources of variation), increasing statistical power, while directly modeling the hierarchical structure of data (participant- and item-level variability)^70^, making the findings robust and not dependent on any idiosyncratic properties of individual items.

We tested if any interaction with Group was significant, which was followed up with post-hoc comparisons separately for each group (with emmeans^71^, two-tailed paired *t*-tests).

The within-group effects investigated comprised identity priming (identity vs. control primes), the mirror cost (identity vs. mirrored), and rotation cost (identity vs. rotated), and whether these effects were modulated by letter type. We hypothesized that dyslexic readers would still show mirror invariance, that is, as fast performance on mirrored as on identity prime conditions (assessed with pairwise comparisons; paired *t*-tests). Thus, to ensure that such null result under NHST (i.e., *p* > 0.05) suggests mirror invariance, we also computed the *Bayes Factor* (BF) using the BayesFactor package^55^, with the default settings for multivariate Cauchy prior distribution. The BF is an odds ratio, where a value of 1 indicates equal evidence for both competing hypotheses. A BF∈ [3, 10 suggests moderate evidence, while BF > 10 indicates strong evidence in favor of the hypothesis in the numerator compared to the one in the denominator^54,72^. Whenever the null hypothesis is favored, we report BF_01_, and BF_10_ when the alternative hypothesis is favored (two-tailed test). Because overall group differences in RTs can artificially inflate interaction terms, we additionally tested the Group × Prime × Letter interaction in an ANOVA on z-transformed RTs, following Faust et al.’s ^73^ rate-and-amount model, which is reported in Supplementary Material.

Second, to directly compare the mirror costs of the two groups while controlling for overall speed, we used a standardized index: *Cohen’s d*^53^, computed for each participant over raw RTs (in ms, for correct word responses; cf. Fernandes et al^19,34^) as the difference between the mean RTs in the identity and in the mirrored-letter prime conditions divided by the pooled *SD* of these conditions. This effect size measure, commonly used in meta-analyses, expresses here the magnitude of a mirror cost in standard deviation units, and hence, is not influenced by between-group differences in speed, thus allowing comparisons across groups and studies^53^. More negative values reflect stronger mirror-image discrimination. These individual *d* values were analyzed in a 2 (Group: controls vs. dyslexics) × 2 (Letter: reversible vs. nonreversible) ANOVA. Following the omnibus test, we ran one-tailed one-sample *t*-tests against zero (as by definition a mirror cost would be negative, justifying a one-tailed test) and between-group comparisons, accompanied by Bayesian analyses (BF₀⁺) to quantify support for the null when appropriate (whenever a result was not significant under NHST, i.e., *p* > 0.05). Effect sizes were reported throughout as η_p_^2^^53^. We thus examined whether the mirror cost was significant (that is, more negative than a null result) and whether the mirror cost of dyslexics was smaller than the mirror cost of controls. Effect sizes of the differences were computed with Cohen’s *d*^53^.

## Results

### Performance on words

For the sake of completeness, the analysis of errors in word trials is reported in Supplementary Material. Both groups had high overall accuracy (*M*_dyslexic_ = 94.15%, *SD* = 3.72; *M*_control_ = 94.31%, *SD* = 4.20). Consistent with previous studies^48^, control and dyslexic college students did not significantly differ on error rates, demonstrating the extensive print experience of high-functioning dyslexic adults.

More important, as shown in Table 2, the analysis run on RTs for correct word responses (3.06% data trimmed) revealed a significant Group x Letter x Prime interaction (confirmed by the ANOVA on z-scores adopting the rate-amount model^73^, reported in Supplementary Material and illustrated in Fig. S1 in Supplementary Material).

**Table 2:** Fixed effect Omnibus tests (Type III Anova) on log RTs in correct word responses.

Fixed-effects	LogRT, linear model^a^
Group	***F*(1, 36.0) = 15.316, ***p* < .001, *MS* = 0.745 *
Letter	*F*(1, 372.6) < 1, *p* = .930
Prime	***F*(3, 13,161.1) = 45.365, ***p* < .001. *MS* = 2.208 *
Group x letter	*F*(1, 13,055.7) = 2.128, *p* = .145, *MS* = 0.104
Group x prime	*F*(3, 13,295.0) < 1, *p* = .516
Letter x prime	*F*(3, 13,160.9) = 2.050, *p* = .105
Group x letter x prime	***F*(3, 13,294.9) = 3.206, ***p* = .022, *MS* = 0.156 *

Omnibus effects were extracted after centering each variable (contr.sum).

^a^ Log(RT) analysis: 13,484 datapoints; 384 items; 38 participants; p-values calculated via Satterthwaite’s approximation. AIC = − 1615.27; BIC = − 1472.594. Model equation (from lme4): lmer(log(RT) ~ ReadGroup * primeC * letterC + (1|Subject) + (1|Item), data = RT_word, control = lmerControl(optimizer = ‘bobyqa’)).

* *p* ≤ .01 (significant effects are in bold).

**Fig. 2:**
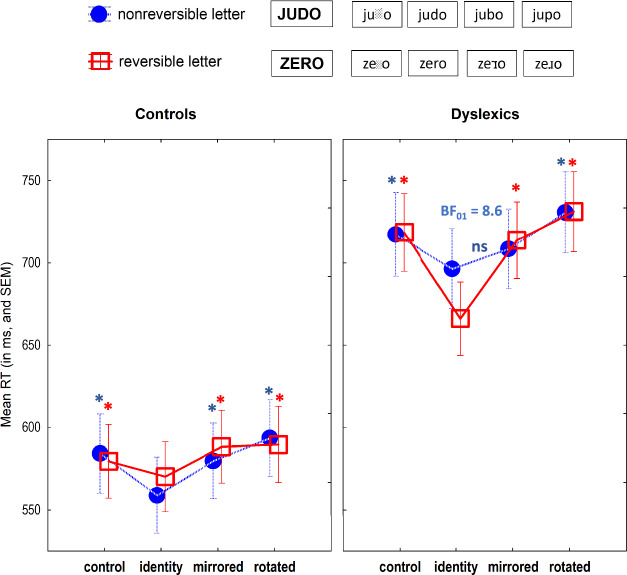
RTs for correct word trials by controls and dyslexics, separately by prime (control, identity, mirrored, rotated) and letter type (nonreversible letter: dashed line, blue circle; reversible letter: full line, red square; online figure in color), with examples of TARGET and primes in each letter set. * Significant differences (*p* < .05) relative to the identity prime condition; ns = nonsignificant difference. BF_01_ = BF for the test of equivalence between the identity vs. mirrored prime conditions.

As shown in Fig. 2, both groups showed a similar pattern of results for reversible letters. They presented identity priming effects: faster decision on words preceded by identity than control primes by typical and dyslexic readers, *t*(13,241) = − 5.43, and *t*(13,422) = − 5.42, respectively, both *p*s < 0.001. Both also showed significant mirror and rotation costs: typical and dyslexic readers were significantly slower on words preceded by mirrored and rotated reversible letters than on words preceded by identity primes; typical readers, *t*(13,155) = −4.05, and *t*(13,088) = −6.14, respectively, and dyslexic readers, *t*(13,422) = −5.42, and *t*(13,107) = − 8.08, respectively, *p*s < 0.001. In short, dyslexics were as sensitive as controls to the orientation differences of letters for which orientation is a diagnostic feature, including when processing mirror images.

In contrast, dyslexics differed from controls only on the mirror cost for nonreversible letters. Both groups presented identity priming effects when processing nonreversible letters: dyslexics, *t*(13,157) = −4.55, *p* < 0.001; controls, *t*(13,422) = −2.56, *p* = 0.011. Dyslexic and control readers also presented significant rotation costs, *t*(13,094) = − 6.14, and *t*(13,422) = −4.29, respectively, both *p*s < 0.001. However, whereas typical readers showed a significant mirror cost, *t*(13,156) = −3.40, *p* < 0.001, dyslexics did not, *t*(13,421) = −1.50, *p* = 0.11. Importantly, there was robust evidence for the equivalence when dyslexic readers processed identity primes and mirrored nonreversible letter primes, BF_01_ = 8.21 (error: 0.29%). Mirror invariance was thus specific for nonreversible letters. In contrast, dyslexics’ mirror cost for reversible letters was robust, BF_10_ = 2543.99 (error: 1.28%).

### Standardized mirror cost

Dyslexic and controls differed only when processing mirror images of nonreversible letters but not plane rotations. To further investigate it, we directly compared dyslexic vs. controls in a mixed Group x Letter ANOVA run on a standardized index of the mirror cost^19,34^, which further confirmed this pattern of results: Group x Letter, *F*(1, 36) = 5.46, *p* = 0.025, η_p_^2^ = 0.132, *MSE* = 0.04 (Group: *F* < 1; Letter: *F*(1, 36) = 4.59, *p* = 0.04, η_p_^2^ = 0.113, *MSE* = 0.04).

**Fig. 3:**
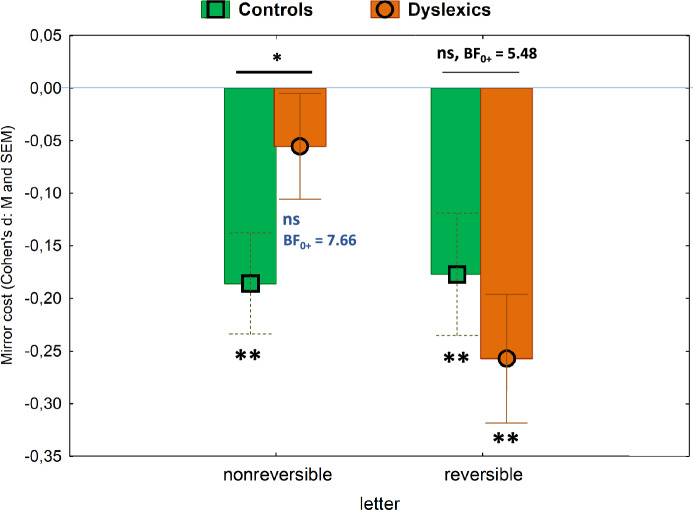
Standardized mirror cost computed as Cohen’s d for nonreversible and reversible letters by controls and dyslexics (green bar and square, and orange bar and circle, respectively; online figure in color). The blue horizontal line corresponds to a null mirror cost (no significant difference on word decisions preceded by mirrored and identity primes). * *p* < .05, ** *p* < .01, ns = nonsignificant difference. BF0 +  = BF for the test of equivalence (one-tailed).

As shown in Fig. 3, dyslexic and typical readers showed significant mirror costs when processing reversible letters, *t*(18) = − 4.67, *d* = 1.10, and *t*(19) = − 2.82, *d* = 0.63, respectively, both *p*s < 0.001. Their mirror costs did not differ from one another, *t* = 0.96, *d* = 0.31, *p* = 0.83, BF_0+_  = 5.48. It was only when processing nonreversible letters that dyslexics showed a significantly smaller mirror cost than typical readers, *t*(36) = −1.87, *d* = 0.61, *p* = 0.035. The same pattern of results was found in the aforementioned ANOVA run on z-scores of RTs^73^ (see in Supplementary Material).

In line with the previous analyses, typical readers showed a significant mirror cost when processing nonreversible letters, *t*(19) = −3.98, *d* = 0.89, *p* < 0.001, which was equivalent to their mirror cost for reversible letters, *t*(19) = −0.14, *d* = 0.03, *p* = 0.14, BF_01_ = 4.26. In contrast, dyslexics presented a significantly smaller mirror cost for nonreversible than for reversible letters, *t*(17) = 3.09, *d* = 0.73, *p* = 0.007, BF_01_ = 0.13. Notably, dyslexics’ mirror cost for nonreversible letters was null, *t*(18) = −1.07, *d* = 0.25, *p* = 0.15, signaling (again) mirror invariance, as supported by Bayesian statistics, BF_0+_  = 7.66.

## Discussion

The present results are clear-cut. Dyslexic college students continue to treat mirror images of nonreversible letters as equivalent, despite years of reading practice and word recognition accuracy comparable to typical readers. These high-functioning dyslexic adults performed similarly to controls when processing reversible letters. However, for nonreversible letters, mirror invariance remained active, indicating that these dyslexic adults continued to be influenced by the original perceptual biases of object recognition.

Like typical readers, dyslexics showed rotation costs for both reversible and nonreversible letters. Thus, both groups automatically discriminate plane-rotations, in line with previous findings in children^39^. However, dyslexics specifically failed to show automatic mirror-image discrimination when processing nonreversible letters. These findings cannot be simply explained by a heightened sensitivity to visual cues by dyslexic readers^49^. They can neither be attributed to general high-level visual difficulties^42^, noisy representations^7^, nor to visual similarity or to the presence of unfamiliar symbols in mirrored or rotated nonreversible-letter primes, because all these accounts would predict similar pattern of results for mirrored and rotated transformations.

For example, if the mere presence of a nonletter symbol in mirrored and rotated primes of nonreversible letters (e.g., ɟ or ɟ instead of f) were disruptive, then these primes should have produced comparable interference. Yet, both typical and dyslexic readers exhibited a rotation cost of similar standardized magnitude. Thus, the finding that dyslexic readers exhibited mirror invariance exclusively for nonreversible letters (i.e., equivalent word recognition in identity and mirrored prime conditions) cannot be plausibly attributed to the presence of a nonletter. This pattern of results challenges any general account. Furthermore, both groups showed identity priming effects for both letter types, which were robust and comparable, indicating effective lexical access and activation in typical and dyslexic adult readers.

Notably, the difference between groups in mirror-image processing of nonreversible letters is not merely quantitative. Bayesian analyses provided strong evidence for equivalence between identity and mirrored primes of nonreversible letters in dyslexics. This result was consistent across all statistical analyses conducted, whereas typical readers showed robust mirror costs—pointing to a qualitative difference. Mirror invariance in developmental dyslexia is not a general visual processing issue, but one specific to mirror images, which is shaped by reading ability.

Previous masked priming studies in different alphabetic languages^19–22^ have consistently shown that neurotypical fluent adult readers exhibit mirror and rotation costs, even for nonreversible letters, suggesting automatic mirror-image discrimination during letter coding. In contrast, the results observed here in dyslexic college students closely resembled those previously reported in 5^th^-grade typical readers^34^, despite the dyslexic group being as accurate in visual word recognition as typical college students. Thus, dyslexic adults behaved like typical readers who still lack reading expertise, indicating that they have not learned from their perceptual experience to the same extent as typical readers. In this sense, “mirror invariance dies hard” in developmental dyslexia: despite years of reading experience, dyslexic college students continue to exhibit residual sensitivity to mirror-image transformations, consistent with reduced and atypical orthographic tuning ^6–9,37,38,50^.

We must acknowledge that dyslexic college students may represent a less severely affected subgroup compared to dyslexic adults who did not pursue higher education. However, their greater reading experience strengthens the conclusion that the present findings cannot be solely attributed to low reading level, reduced print experience, or comorbid conditions^7,43^. While these findings may not fully generalize to all dyslexic adults, readers of the Latin alphabet, dyslexic college students nonetheless represent a conservative test case. They are presumably among the most experienced and/or better compensated readers within the adult dyslexic population. Therefore, the persistence of residual mirror invariance for nonreversible letters suggests that this phenomenon may be more widespread among adults with developmental dyslexia and could extend to reversible letters in those with less reading experience. This conclusion converges with previous findings showing that dyslexic adults exhibit greater difficulty in discriminating mirrored reversible letters compared to other visually similar pairs (e.g., “cod—cob” vs. “fire – tire”) in same-different matching or letter naming tasks^37,44,47,51,52^. In typical readers, automatic mirror-image discrimination for nonreversible letters emerges only around the end of Grade 6 and is linked to the consolidation of abstract letter representations^34,40^. It is thus likely that dyslexic adults with less reading experience still process reversible and nonreversible letters in a mirror-invariant manner^44,47,51,52^. The present results contribute by demonstrating that this effect pertains to deviant orthographic processing.

The difficulty in mirror-image discrimination reflects a learning deficit rooted in the conflict between the original mirror invariance of object recognition and the mirror-image discrimination required by the written code^1,5,11,35^. Readers become sensitive to the critical features and structural relations that distinguish letters, including mirror-image discrimination, which in turn shapes how letters are represented in perceptual space^5,17,19^. For reversible letters, orientation contrasts (e.g., p, q, d) correspond to different letters, so misorientations activate multiple letter candidates. Furthermore, as reversible letters are part of different words (e.g., pig, dig, big), mirror-image discrimination is reinforced at lexical, besides prelexical, level^19^. This likely explains why automatic mirror-image discrimination emerges earlier for these letters in typical reading development^34^ and why this pattern was also observed here in high-functioning dyslexic adults. In line with the proposal of Fernandes et al^15,19,34,39^, the likelihood that changes in orthographic dynamics generalize to symbols that are not subject to such pressure (here mirrored and rotated nonreversible letters; e.g., r and ɹ) depends on the quality of letter representations, which are deficient in dyslexia^6–8,37,38,47–50^, and responsible for the pattern of results found in the present study. Our interpretation thus contrasts with former perspectives that viewed reversal errors as a cause of the reading disorder (for a review, see^39^). Instead, these errors reflect an orthographic deficit, which is one of the underlying causes of dyslexia.

The educational implication that follows is that, although mirror-invariance remains active during orthographic processing in dyslexia, extensive reading experience may help consolidating more precise letter representations, ultimately supporting automatic mirror-image discrimination of letters for which orientation is functionally relevant, leading to more efficient word recognition.

The masked priming paradigm adopted here is the most effective tool for testing orthographic coding in ways less likely to be influenced by extraneous processes (including phonological ones) or strategic factors^18^. Thus, the difficulties dyslexic adults experience with mirrored letters cannot be attributed to a phonological deficit or to explicit difficulties in processing orientation. Instead, they are more likely the result of insufficient print tuning and a lack of reading expertise^45,46^, as evidenced by hypoactivation in the left vOT and deviant letter processing^6–8,37,38,47–50^.

Note, however, that the similar behavioral results of dyslexic and typical readers when processing reversible letters do not guarantee that the underlying neural mechanisms are the same. Given the atypical neural activation in orthographic processing by dyslexic adults (including college students)^6–8^, future studies with high-resolution neuroimaging methods should determine whether this apparent behavioral normalization reflects indeed the same processing route.

The fact that orientation transformations of nonreversible letters are not real letters but are automatically discriminated by typical readers corresponds to a *transfer effect*: a generalization of mirror-image discrimination to nonletters, at least as long as they are embedded in letter strings. Several well-known brands (e.g., Desigual, SONY VAIO) deliberately employ mirrored, rotated, or geometrically altered letters or shapes in their logos and visual identities, capitalizing on the fact that (typical) readers are aware of their similarity while able to discriminate these nonleters. These stylizations, which are legible and even aesthetically appealing in real-world contexts, leverage our visual system’s tolerance for and sensitivity to orientation transformations, including mirror images. The absence of such transfer effect in dyslexic college students is consistent with previous findings of reduced transfer effects in audiovisual perceptual learning tasks in dyslexia^74,75^.

While the purpose of our study was not to determine whether any theoretical account of developmental dyslexia could explain the present findings, the recent proposal of Behrmann and colleagues^45,46^ seems promising. It suggests that the reading deficit may stem from perceptual learning with limited transfer effects, as the plasticity processes driven by reading experience in the left vOT are less efficient in individuals with developmental dyslexia. Kershner^76^ also proposed that dyslexia could stem from reduced neuroplasticity in specific brain regions recruited for reading, which would result in (apparent) domain-specific impairments, particularly affecting a cultural acquisition like reading. Given the bidirectional influences between object recognition and reading^1,2,5,11,77,78^, reduced plasticity in the vOT would affect not only reading itself but also any transfer effect. Note that the left vOT is the neural locus of mirror-image discrimination during letter processing whereas it responds in a mirror invariant manner to other (nonlinguistic) visual categories^23,24^. Furthermore, when 13-year-old dyslexics were trained in reading nonwords in a novel zig-zag graphemic format, with control readers matched to the same initial performance level to rule out differences in letter coding or phonological decoding, dyslexics showed slower learning rate and also failed to exhibit the transfer effect seen in controls when reading a new zig-zag list after training^75^. Note that this perceptual learning problem and reduced transfer effect happens only in specific conditions. For example, dyslexic adults showed smaller transfer effects than typical readers for audio-visual pairing of letters but not for motor-visual pairing^74^. These findings also align with evidence that dyslexic adults show delayed activation of orthographic information and outside the left vOT^6^. Relative to control readers, both dyslexic children and adults present a deficiency in a neurochemical correlate of brain density and function that supports and maintains myelination, that is, total N-acetylaspartate (tNAA) in the visual cortex^79^. Reduced plasticity in the vOT could thus explain why dyslexic college students showed mirror invariance when processing nonreversible letters, whose automatic mirror-image discrimination corresponds to a transfer effect.

Whether the difficulty observed here in mirror-image discrimination can be explained by a deficit in high-level visual processing in dyslexia remains to be determined. Due to the bidirectional interplay between object recognition and reading, there is an inherent chicken-and-egg problem in this possibility, as discussed by Kristjánsson and Sigurdardottir^42^. Indeed, considering that even rudimentary reading skills are sufficient to trigger changes in visual processing of other categories, and that letter knowledge acquired prior to formal literacy instruction contributes to the beginning of these changes^2,13,15,77,80^, any differences between dyslexic and typically-developing participants–including “abnormalities in the ventral visual pathway”^42^ (p. 9) and (any) “specific visual-spatial talent”^81^ (p. 427)–might result from the limited bidirectional influences of reading and object recognition in developmental dyslexia. In other words, unlike typical readers, who show changes in high-level visual processing as their reading skills develop^77,78,80^, dyslexic readers may still exhibit the ‘baseline’ visual processing found in non-readers. These so-called “high-level visual difficulties” would thus be relatively specific and linked to problems in achieving reading expertise. The relationship between reading and visual processing of other categories thus requires further specification and future research, including with illiterate adults and longitudinal research with typical and dyslexic readers.

## Supplementary Information

Supplementary Information.

## Data Availability

Raw data, statistical analyses code, and additionally materials are publicly available at https://osf.io/z38w9/.
