# Supplementary material for: Mirror invariance dies hard during letter processing by dyslexic college students
Source: Sci Rep. 2025 Oct 27;15:37395. doi: 10.1038/s41598-025-21092-5 (PMC12559734; doi:10.1038/s41598-025-21092-5)
Supplement: Supplementary file 1 — Supplementary Information. [file 41598_2025_21092_MOESM1_ESM.docx]

**Supplementary Material**

**Mirror invariance dies hard during letter processing by dyslexic college students**

**Tânia Fernandes^*^, Mariona Pascual, & Susana Araújo**

Faculty of Psychology, Universidade de Lisboa, Alameda da Universidade, 1649-013 Lisbon, Portugal.

* Corresponding author: [taniapgfernandes@gmail.com](mailto:taniapgfernandes@gmail.com), [tpfernandes@psicologia.ulisboa.pt](mailto:tpfernandes@psicologia.ulisboa.pt).

**Table S1.** *Mean performance (RTs for correct responses in ms; errors in %) in nonword trials by each group.*

| **Group** |  | **Letter** | | | | | |
| --- | --- | --- | --- | --- | --- | --- | --- |
|  |  | **nonreversible** | |  | | **reversible** | |
|  | **prime** | *RTs (ms)* | *Errors (%)* |  | *RTs (ms)* | | *Errors (%)* |
|  |  |  |  |  |  | |  |
| Controls | control | 641 (102) | 7.81 (12.64) |  | 644 (94) | | 10.00 (12.66) |
|  | Identity | 644 (109) | 8.44 (13.37) |  | 639 (94) | | 8.75 (13.29) |
|  | mirrored | 640 (105) | 8.44 (11.91) |  | 639 (96) | | 8.65 (11.21) |
|  | rotated | 637 (99) | 8.23 (11.87) |  | 645 (89) | | 8.33 (11.96) |
|  |  |  |  |  |  | |  |
| Dyslexics | control | 852 (137) | 11.11 (9.69) |  | 867 (148) | | 12.04 (9.39) |
|  | identity | 848 (152) | 12.27 (10.43) |  | 867 (139) | | 16.09 (11.41) |
|  | mirrored | 861 (140) | 12.38 (10.49) |  | 869 (133) | | 11.81 (12.52) |
|  | rotated | 864 (139) | 12.96 (9.73) |  | 869 (149) | | 14.50 (12.40) |

*Note.* *SD* is in parenthesis (derived from data aggregated by participants).

**Table S2.** *Mean errors (in %) on word trials by each group.*

| **Group** |  | **Letter** | | | | | |
| --- | --- | --- | --- | --- | --- | --- | --- |
|  |  | **nonreversible** | |  | | **reversible** | |
|  | **prime** |  |  |  |  | |  |
|  |  |  |  |  |  | |  |
| Controls | control | 5.42 (4.80) | |  | 4.58 (3.86) | | |
|  | Identity | 4.38 (4.48) | |  | 4.38 (4.32) | | |
|  | mirrored | 5.00 (4.45) | |  | 6.35 (6.81) | | |
|  | rotated | 7.81 (6.41) | |  | 8.13 (6.00) | | |
|  |  |  |  |  |  | |  |
| Dyslexics | control | 5.90 (4.36) | |  | 4.75 (2.75) | | |
|  | identity | 6.25 (3.10) | |  | 4.51 (2.18) | | |
|  | mirrored | 5.79 (4.66) | |  | 6.71 (5.03) | | |
|  | rotated | 8.56 (7.69) | |  | 5.67 (3.26) | | |

*Note.* *SD* is in parenthesis (derived from data aggregated by participants).

**Analysis on errors in word trials**

We used R (R Team, 2020) and the R packages lme4 (Bates et al., 2015) and lmerTest (Kuznetsova et al., 2022) to fit a *generalized linear mixed model* to error rate (binomial distribution) with group, letter, and prime as fixed-effect factors (sum coding of all factors), and with by-participant and by-item random intercepts (formula: glmer(ERROR ~ ReadGroup * primeC * letterC + (1|Subject) + (1|Item), data = ACC_word, family = 'binomial', control = glmerControl(optimizer="bobyqa", optCtrl=list(maxfun=2e5)))). Significance of fixed effects was assessed with LRT method (Likelihood Ratio Test).

As shown in Table S2, both groups presented low error rates. The mixed model ANOVA (Type III, with LRT method) showed that there was no significant difference between dyslexic and control readers on mean error rate, *X^2^*_(1)_ = 0.24, *p* = .625. The main effect of prime, *X^2^*_(3)_ = 28.56, *p* < .001 was the only significant effect found; main effect of letter, *X^2^*_(1)_ = 0.42, *p* = .52; Group x Letter, *X^2^*_(1)_ = 3.13, *p* = .08, Group x Prime, *X^2^*_(1)_ = 3.88, *p* = .28, Letter x Prime, *X^2^*_(3)_ = 5.77, *p* = .12, Group x Letter x Prime, *X^2^*_(3)_ = 0.83, *p* = .84.

**Analysis on z-scores in word trials**

We used R (R Team, 2020) and the R packages afex (Singmann et al., 2017) and emmeans (Lenth, 2020) to run a mixed ANOVA on the *z-*score transformation of RTs (in ms) in correct word trials, adopting the rate-amount model (Faust et al., 1999), with group (between-participants), and letter and prime types (within-participant) as factors. This analysis ensured the Group x Letter x Prime interaction found in the LMM analysis was not merely due to over-additivity effects (see main text).

The main effect of prime was significant, *F*(3, 108) = 33.16, *p* < .001, *MSE* = 0.67, η_p_^2^ = .479, demonstrating that the one-character manipulation of the prime significantly affected visual word recognition overall (Prime x Letter, *F*(3, 108) = 1.32, *p* = .27). More important, the three-way interaction between group, letter, and prime also remained significant, *F*(3, 108) = 2.88, *p* = .039, *MSE* = 0.89, η_p_^2^ = .074, as shown in Figure S1 (Group x Letter, *F*(1, 36) = 1.38, *p* = .25; all other *F*s < 1).

To better investigate this three-way interaction, we first examined separately in each group if the Letter x Prime interaction was significant. As shown in Figure S1, for control readers the significant effect of prime, *F*(3, 36) = 31. 43, was not modulated by letter type, *F* < 1 (main effect of letter type, *F* < 1), whereas for dyslexic readers the effect of prime, *F*(3, 36) = 32.40, *p* < .001, was significantly modulated by letter type, *F*(3, 36) = 5.27, *p* = .004.

**Figure S1.** *Mean z-scores (and SEM as error bars) in each prime condition for reversible and nonreversible letters (red and blue lines, respectively) in word trials by each group.*


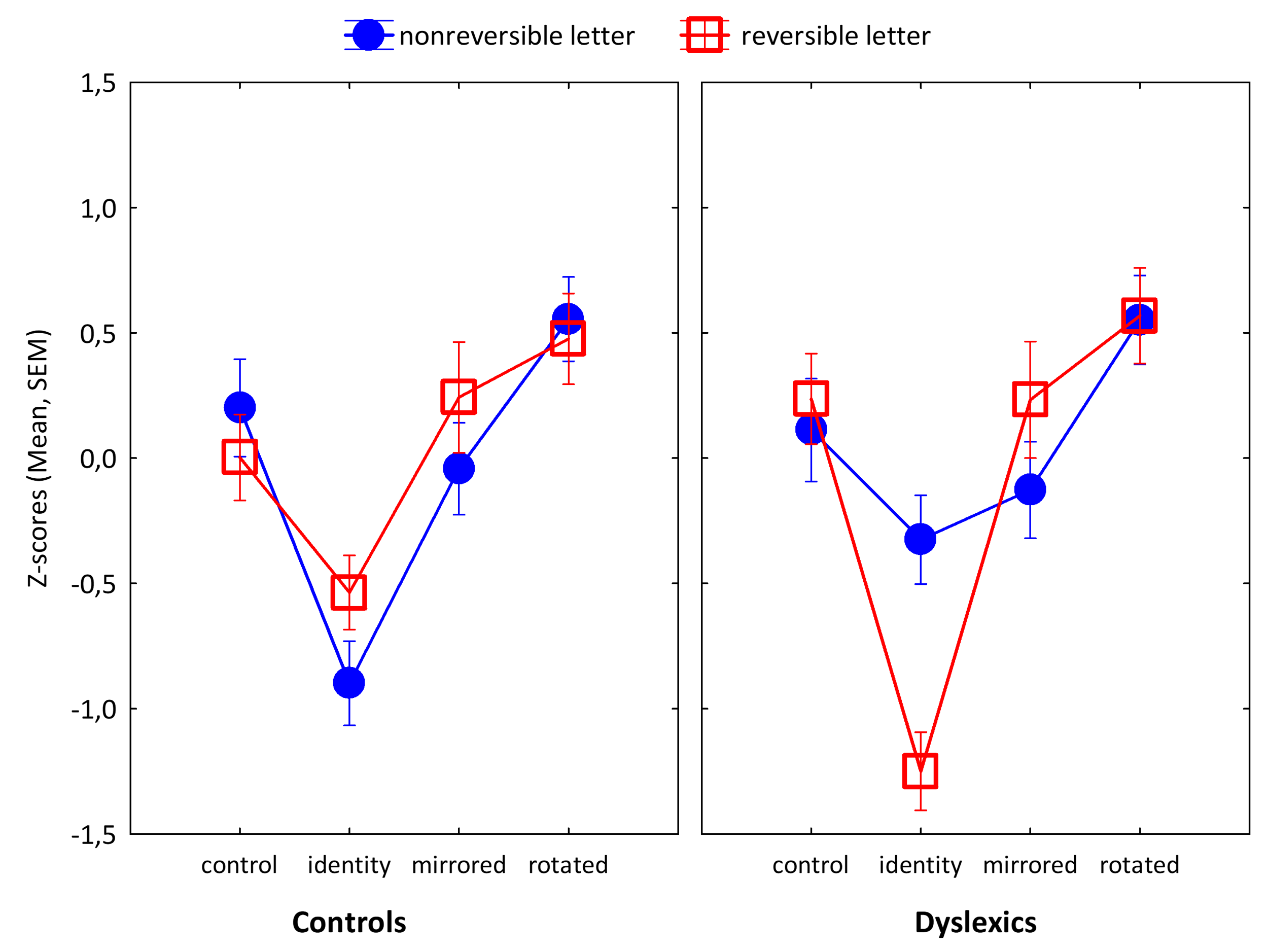


As shown in Figure S1, identity priming was significant, with faster word decisions in identity than control conditions for nonreversible and reversible letters, *F*(1, 36) = 12.89 and = 31.12, respectively, both *p*s ≤ .001, of similar magnitude for dyslexic and control readers: no significant difference were found between groups for nonreversible and reversible letters, *F*(1, 36) = 2.46, and = 2.67, both *p*s ≥ .11.

For reversible letters, as in the analysis run with LMM on RTs reported in the main text, the pattern of results of dyslexic readers was similar to the one of typical readers. Both groups showed significant mirror costs, that is, significantly slower decision on target words preceded by mirrored-letter primes than identity primes: typical readers, *t* = 2.69, *p* = .01, 95% CI [0.19, 1.37]; dyslexic readers, *t* = 4.85, *p* < .001, 95% CI [0.86, 2.10]. This mirror cost for reversible letters was of similar magnitude for control and dyslexic readers, *t* = -1.67, *p* = .10. Likewise, both groups showed significant rotation costs when processing reversible letters, with slower performance on words preceded by rotated-letter primes than identity primes: typical readers, *t* = 4.03, *p* < .001, 95% CI [0.50, 1.52]; dyslexic readers, *t* = 6.88, *p* < .001, 95% CI [1.28, 2.35], which were of similar magnitude (typical readers vs. dyslexic; Bonferroni correction, *t* = -2.211, *p* = .20). In short, dyslexics were as sensitive as control readers to the orientation differences of reversible letters, including when processing mirror images.

In contrast, for nonreversible letters, whereas typical readers showed the same pattern of performance as the one found for reversible letters, dyslexics showed a significant difference when processing mirror-images of nonreversible letters. Specifically, whereas typical readers showed a significant mirror cost, with slower decision on words preceded by mirrored nonreversible letter than identity primes, *t* = 3.42, *p* < .002, 95% CI [0.35, 1.36], dyslexic readers were as fast on word targets preceded by identity as by mirrored nonreversible letter primes, *t* = 0.75, *p* = .46, 95% CI [-0.34, 0.73]. Note, however, that as aforementioned, dyslexic readers showed significant identity priming effects when processing nonreversible letters. Furthermore, dyslexic readers also showed a significant rotation cost, with slower word decisions for targets preceded by rotated nonreversible letter than identity primes, *t* = 3.42, *p* = .002, 95% CI [0.36, 1.40], just like typical readers did, *t* = 5.97, *p* < .001, 95% CI [0.96, 1.95]. Indeed, the magnitude of this rotation cost for nonreversible letters was similar in dyslexic and control readers, *t* = 1.63, *p* = .67, and hence, there was no reason to believe that dyslexics had any difficulty when processing plane rotations of either reversible or nonreversible letters.

**References**

Bates, D., Mächler, M., Bolker, B., & Walker, S. (2015). Fitting Linear Mixed-Effects Models Using lme4. *Journal of Statistical Software*, *67*(1). <https://doi.org/10.18637/jss.v067.i01>

Faust, M. E., Balota, D. A., Spieler, D. H., & Ferraro, F. R. (1999). Individual differences in information-processing rate and amount: Implications for group differences in response latency. *Psychological Bulletin*, *125*(6), 777-799. <https://doi.org/10.1037/0033-2909.125.6.777>

Kuznetsova, A., Brockhoff, P. B., & Christensen, R. H. B. (2022). lmerTest Package: Tests in Linear Mixed Effects Models [denominator degree of freedom, Satterthwaite's approximation, ANOVA, R, linear mixed effects models, lme4]. *Journal of Statistical Software*, *82*(13), 26. <https://doi.org/10.18637/jss.v082.i13>

Lenth, R. (2020). *emmeans: Estimated Marginal Means, aka Least-Squares Means. R package version 1.4.7.* In <https://CRAN.R-project.org/package=emmeans>

Singmann, H., Bolker, B., Westfall, J., & Aust, F. (2017). *afex: Analysis of Factorial Experiments*. In <https://CRAN.R-project.org/package=afex>
